# Deep Unsupervised Domain Adaptation with Time Series Sensor Data: A Survey

**DOI:** 10.3390/s22155507

**Published:** 2022-07-23

**Authors:** Yongjie Shi, Xianghua Ying, Jinfa Yang

**Affiliations:** School of Artificial Intelligence, Peking University, No. 5 Yiheyuan Road, Haidian District, Beijing 100871, China; shiyongjie@pku.edu.cn (Y.S.); jinfayang@pku.edu.cn (J.Y.)

**Keywords:** deep learning, unsupervised domain adaptation, time series sensor data, survey

## Abstract

Sensors are devices that output signals for sensing physical phenomena and are widely used in all aspects of our social production activities. The continuous recording of physical parameters allows effective analysis of the operational status of the monitored system and prediction of unknown risks. Thanks to the development of deep learning, the ability to analyze temporal signals collected by sensors has been greatly improved. However, models trained in the source domain do not perform well in the target domain due to the presence of domain gaps. In recent years, many researchers have used deep unsupervised domain adaptation techniques to address the domain gap between signals collected by sensors in different scenarios, *i.e.*, using labeled data in the source domain and unlabeled data in the target domain to improve the performance of models in the target domain. This survey first summarizes the background of recent research on unsupervised domain adaptation with time series sensor data, the types of sensors used, the domain gap between the source and target domains, and commonly used datasets. Then, the paper classifies and compares different unsupervised domain adaptation methods according to the way of adaptation and summarizes different adaptation settings based on the number of source and target domains. Finally, this survey discusses the challenges of the current research and provides an outlook on future work. This survey systematically reviews and summarizes recent research on unsupervised domain adaptation for time series sensor data to provide the reader with a systematic understanding of the field.

## 1. Introduction

Sensors capture physical parameters from the observed environment and convert them into observable electrical pulses [1,2,3,4]. A wide variety of sensors are used in a wide range of applications in manufacturing and machinery [5,6,7], transportation [8,9,10,11,12,13], healthcare [14,15,16,17,18] and many other aspects of our daily lives. In the field of mechanical engineering, for example, accelerometer sensors placed around gearboxes or bearings can capture the vibration signals of a machine and predict possible impending failures [7]. In the healthcare field, voltage signals from a patient’s brain are captured by placing voltage sensors on the patient’s brain and used to identify patient commands [16], etc. By continuously recording physical parameters over a period of time, the current operating state of the monitored system can be analyzed and unknown risks can be assessed.

In recent years, the analysis techniques for time series data have been rapidly developed. Commonly used models for processing time series data are spectral and wavelet analysis [19,20,21], 1D Convolutional Neural Networks (CNNs) [22,23,24], Recurrent Neural Networks (RNNs) represented by Long Short-Term Memory (LSTM) networks [25,26,27] and Gated Recurrent Unit (GRU) [28,29], and the recently emerged Transformer [30]. Deep models have a large number of parameters and powerful feature extraction capabilities, and the parameters of the models are optimized and updated by loss functions and a gradient back propagation [31,32]. However, deep models are often based on the assumption that the distribution of training and test data is similar. In practice, however, this requirement is not satisfied. For example, in the fault diagnosis of rolling bearings, the vibration signals collected by accelerometer sensors under different loads (e.g., rotation speed) have different patterns. If vibration signals collected under one operating condition are used to train a deep network and data collected under another load condition is used to test the trained model, the performance of the model may be significantly degraded. As another example, in the brain-computer interface, a model trained with a large number of signals collected on one person is tested on another person and the model does not perform well. This is because the performance of the model tends to degrade when the model is trained on one scene (source domain) and tested on data collected on another scene (target domain). One way to solve this problem is to label a large amount of labeled data in a new scene and retrain the deep model. However, relabeling the data for each new scene requires significant human resources. Due to the ability to easily obtain unlabeled data in the target domain, many researchers have adopted unsupervised domain adaptation (UDA) techniques to improve the performance of the model in the target domain. That is, the model is trained using labeled data in the source domain and a large amount of unlabeled data in the target domain.

This survey collects papers on unsupervised domain adaptation with time series sensor data from academic studies in 2018 to date. First, this survey divides them by industry, listing the background of different applications, sensors often used, the sources of data discrepancies, and the commonly used research datasets. Then, this survey classifies these different methods for unsupervised domain adaptation for time series data, including domain adaptation methods aligned in the input space, feature space, and output space, and model-based domain adaptation methods. After that, the paper classifies the existing domain adaptation settings according to the number of sources and target domains and analyzes their advantages. Finally, the survey summarizes the existing methods and looks into possible future research directions. Unlike previous reviews [33,34,35] that focus on a particular research direction, this paper takes sensors that collect time series data as a starting point and surveys different industries that need to monitor the status of their systems and summarizes the sensors they use and the reasons why domain gaps exist. A classification of deep learning approaches to address these domain gaps is presented, as well as the advantages of different adaptation settings. The aim is to provide the reader with a comprehensive and systematic understanding of deep unsupervised domain adaptation for time series sensor data.

**Figure 1:**
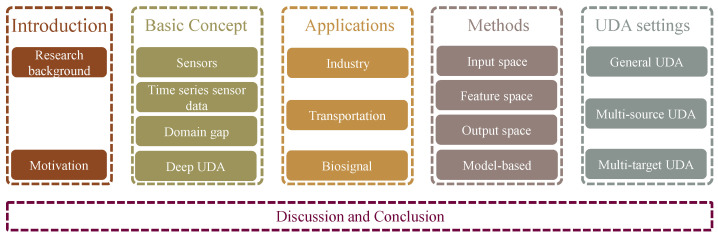
The organization of this survey.

The remainder of the paper is structured as follows (see Figure 1). Section 2 explains some basic concepts. Section 3 divides the collected papers by industry. Section 4 divides the studies according to different adaptation methods. Section 5 divides the existing methods by domain adaptation settings. Section 6 discusses the present study and provides an outlook. Section 7 makes a conclusion. 

## 2. Basic Concept

To facilitate the understanding of this paper, this section explains some basic concepts, including what a sensor is, the definition of time series sensor data, what a domain is, and what domain adaptation and deep unsupervised domain adaptation are.

### 2.1. Sensors

A sensor converts physical phenomena into a measurable digital signal, which can then be displayed, read, or processed further [1]. According to the physical characteristics of sensor sensing, common sensors include temperature sensors that sense temperature, acceleration sensors that sense motion information, infrared sensors that sense infrared information, etc. According to the way of sensing signals, sensors can be divided into active sensors and passive sensors. Active sensors need an external excitation signal or a power signal. On the other hand, passive sensors do not require any external power and produce an output response. LiDAR is an example of an active sensor, as it requires an external light source to emit a laser. By receiving the returned beam, the time delay between emission and return is calculated to determine the distance to an object. Passive sensors, such as temperature sensors, acceleration sensors, and infrared sensors, do not require external excitation and can directly measure the physical characteristics of the system being monitored. A wide variety of sensors are used in different industries and have greatly increased the productivity of society.

### 2.2. Time Series Sensor Data

The time series of length *n* observed by the sensor can be expressed as

(1) $$x=[x_{1},x_{2},\cdots ,x_{t},\cdots ,x_{n}],$$

where the data point $x_{t}$ is the data observed by the sensor at moment *t*. When x is univariate time series data, $x_{t}$ is a real value and $x_{t}\in R$. When x is multivariate time series data, $x_{t}$ is a vector and $x_{t}\in R^{d}$, where the *d* indicates the dimension of $x_{t}$. Much of the time series data collected in practical applications are multivariate data that may be obtained from multiple attributes of a sensor or multiple sensors. For example, in the fault diagnosis of rolling bearings, an acceleration time series of three axes XYZ can be obtained simultaneously by a single accelerometer. Another example is in fault diagnosis of power plant thermal system, where multidimensional time series data are obtained simultaneously by using multiple sensors, such as temperature sensors, pressure sensors, flow rate sensors, etc.

### 2.3. Domain Gap

**Figure 2:**
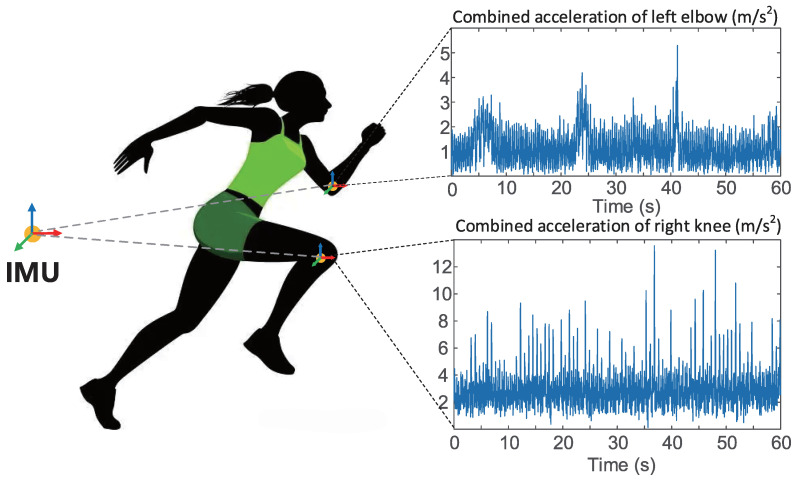
Diagram of human activity recognition based on inertial measurement unit (IMU) [37]. Two IMUs are placed on the arm and leg respectively to record the motion pattern at each position for further recognition of human motion. As can be seen from the figure, there are some differences in the data collected from different limbs. Reprinted with permission from Ref. [37]. Copyright 2020 IEEE.

**Figure 3:**
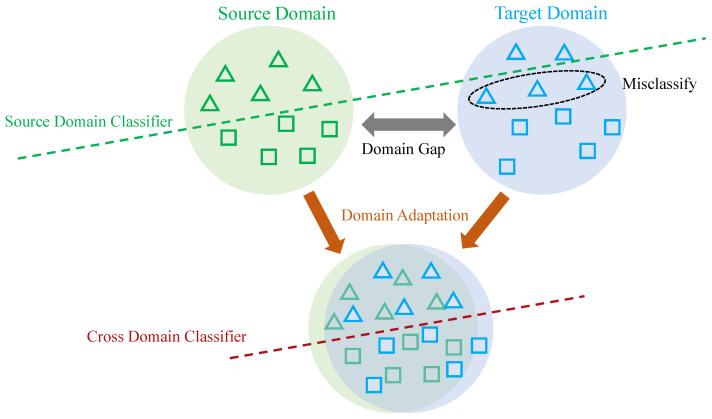
Illustration of source and target data with original feature distributions (**top**), and new features distributions (**bottom**) after domain adaptation, where domain adaptation techniques help to alleviate the “domain shift” problem between source and target domains.

Data collected under a certain distribution is referred to as a domain, and this distribution can be understood as a specific data collection scenario [36]. For example, in human behavior recognition, inertial sensor data collected at the arm is referred to as one domain, while inertial sensor data collected at the leg is referred to as another domain. The data collected in different scenarios differ in distribution, and this difference is known as the domain gap (see Figure 2 and Figure 3).

### 2.4. Deep Unsupervised Domain Adaptation

In the concept of domain adaptation, scholars usually refer to the training set as the source domain and the test set as the target domain. Assuming that the model is trained with data collected at the arm (source domain) and later tested with data collected at the leg (target domain), the model does not perform as well. This is because the deep learning model can only recognize test data with the same distribution as the training data. One way to improve the performance of the model in the target domain is to label a large amount of data in the target domain and fine-tune the model trained in the source domain using a supervised approach. However, labeling a large amount of data requires significant human resources.

Domain adaptation (see Figure 3) refers to adapting the model trained in the source domain to the target domain, i.e., reducing the domain gap between the source and target domains and improving the performance of the target domain. Domain adaptation includes semi-supervised domain adaptation and unsupervised domain adaptation. Semi-supervised domain adaptation requires that samples from the target domain are partially labeled. In contrast, unsupervised domain adaptation does not require the target samples to have labels. Deep unsupervised domain adaptation specifically refers to improving the adaptation ability of deep learning models in the target domain. Due to the complexity and nonlinearity of deep learning models, a large number of algorithms for deep unsupervised domain adaptation have emerged in recent years, including mapping-based algorithms, adversarial learning-based algorithms, etc.

## 3. Applications

Although there are many papers dedicated to UDA of time series sensor data, they have different application backgrounds and research directions. This section systematically analyzes the applications of UDA with time series sensor data in recent years and broadly divides them into three application areas, namely industry, transportation, and biosignal. Each field contains several specific application directions. Moreover, this section summarizes in detail which sensors are included in each application direction, what causes the domain gaps, and which datasets are publicly available. The overall classification results are shown in Table 1, and this section will present them in detail in different subsections.

### 3.1. Industry

This section introduces several applications of UDA in industry, including fault diagnosis of rolling bearings, fault diagnosis of power plant thermal systems, and diagnosis of ball screw faults.

#### 3.1.1. Fault Diagnosis of Rolling Bearings

**Background**. A rolling bearing is a precision mechanical component that changes the sliding friction between the running shaft and the shaft seat, thereby reducing frictional losses [128,129,130,131]. Rolling bearings are a widely used mechanical component in industry and often have extremely demanding performance standards. Machine failures can be catastrophic and lead to expensive downtime. Without effective diagnostics, one cannot make reliable predictions about when a failure will occur. Therefore, effective fault diagnosis is essential in the industry.

**Sensors**. Common types of sensors used for rolling bearing signal acquisition are accelerometer sensors [48,52] as well as microphones [46]. Accelerometer sensors measure the health of machinery by being fixed directly near the machinery to collect the vibration signals generated by the mechanical vibrations. Microphones, on the other hand, capture the operating conditions of machinery by collecting the sound waves generated by mechanical vibrations. The signal collected by the microphone will contain more noise than the accelerometer sensor.

**Domain Gap**. The reasons for the inconsistent distribution of training and test data come from three main sources: different working conditions, different locations of sensors, and different machines.

(1) *Different Working Conditions.* Due to the influence of speed, load, temperature, etc., working conditions often vary during the monitoring period. Collected signals may contain domain shift, which means that the distribution of data may differ significantly under different working conditions. The aim of deep unsupervised domain adaptation-based fault diagnosis is that the model trained using signals under one working condition can be transferred to signals under another different working condition.
(2) *Different Locations of Sensors.* Since sensors installed on the same machine are often responsible for monitoring different components, sensors located near the fault component are more suitable to indicate the fault information. However, key components have different probabilities of failure rates, leading to the situation where signals from different locations have different numbers of labeled data. The aim of unsupervised domain adaptation-based fault diagnosis is that the model trained with plenty of labeled data from one location can be transferred to the target domain with unlabeled data from other locations.
(3) *Different Machines.* For testing cost and safety reasons, it is difficult to collect sufficient samples of marked failures from test machines. In addition, sufficient labeled data can be generated from dynamic simulations or fault seeding experiments. However, the distribution of data from dynamic simulations or fault seeding experiments is different from that of real machines due to similar structure and measurement situations, which is one of the sources of the domain gap.

**Datasets**. Some common open-source rolling bearing fault diagnosis datasets are the Case Western Reserve University (CWRU) Dataset [38] and the Paderborn University (PU) Dataset [39].

**Figure 4:**
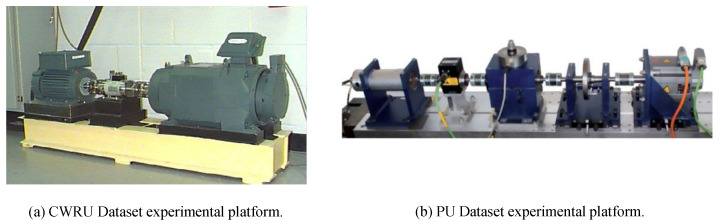
Experimental platforms for CWRU dataset [38] and PU dataset [39]. Reprinted with permission from Ref. [38]. Copyright 2015 Elsevier.

(1) *CWRU* [38]. The CWRU dataset provided by the Case Western Reserve University Bearing Data Center is one of the most famous open-source datasets in intelligent fault diagnosis. CWRU contains one normal bearing and three fault types, including inner fault, ball fault, and outer fault, and is classified into ten categories (one health state and nine fault states) according to different fault sizes. Moreover, CWRU consists of four motor loads corresponding to four operating speeds. The experimental platform of the CWRU dataset can be seen in Figure 4a.
(2) *PU* [39]. PU bearing datasets were provided by Paderborn University (PU) for bearing fault diagnosis based on vibration signal. The PU dataset contains data for variable operating conditions. For example, different radial forces on the bearings, different load torques on the drive system, and different speeds of the drive system. The vibration signals are obtained at a sampling rate of 64 kHz under three types of bearings: realistically damaged bearings, artificially damaged bearings, and healthy bearings. Artificial damages arise in the inner race or outer race, and realistic damages arise in the form of pitting or plastic deformation. The experimental platform of the PU dataset can be seen in Figure 4b.
(3) *IMS* [40]. The IMS dataset is from Prognostics Center Excellence through the prognostic data repository contributed by Intelligent Maintenance System (IMS), University of Cincinnati. The experiment performs bearing run-to-failure tests under constant loads on a specially designed test rig. The rotation speed is 2000 r/min and the sampling frequency is 20 kHz. The tests with outer race failure are investigated and data from different life cycle stages are considered, including severe failure, moderate failure, outer raceway incipient failure, and healthy condition.

#### 3.1.2. Fault Diagnosis of Power Plant Thermal System

**Background**. Power units are developing towards large scale, integration, and complexity. The thermal system is one of the most critical components of a large-scale power unit [132]. In recent years, renewable energy sources such as solar energy and wind energy have been integrated into the grid [133]. Due to their stochastic and intermittent nature, the task of frequency and peak regulation still needs to be performed by coal-fired power plants. This may lead to harsher operating conditions for the units and a higher probability of failure. Therefore, accurate fault diagnosis of thermal systems is an urgent problem that needs to be addressed.

**Sensors**. The monitoring of thermal power unit operating conditions includes many physical parameters, such as the temperature and pressure of high-pressure heaters, and the flow rate of condensate, etc. Therefore, the commonly used sensors in the fault diagnosis of the power plant thermal system are temperature sensors, pressure sensors, flow rate sensors, etc.

**Domain Gap**. In the fault diagnosis of the power plant thermal system, the domain gap comes from two main areas.

(1) *Fault Severity*. For example, for feedwater leak faults, different leak volumes represent different levels of severity [60,61]. Although they are all fault data, the data collected from them differ in distribution.
(2) *Different Load Conditions*. The distribution of the fault data collected by the power plant thermal system is different under different load conditions [60,61], which leads to the domain shift.

#### 3.1.3. Diagnosis of Ball Screw Failure

**Background**. Ball screws are important mechanical devices capable of converting rotary motion into high-precision linear motion, and they are widely used in feed drive systems for machine tools [134]. Ball screws have important accuracy tolerances, and even slight degradation of the components can increase the operational risk. For example, the preload level of ball screws is carefully calibrated to maximize the expected life of the ball screw without affecting the repeatability of the motion. However, with use, a reduction in preload reduces the stiffness of the ball screw assembly and may eventually lead to a loss of positional accuracy. Therefore, it is important to accurately and reliably identify the health of the ball screw.

**Figure 5:**
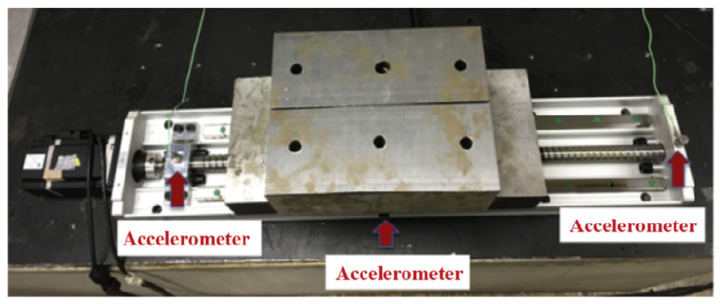
The ball screw test rig [63]. Reprinted with permission from Ref. [63]. Copyright 2020 Elsevier.

**Sensors**. During the operation of ball screws, vibration signals are often used to determine if a ball screw is faulty. Therefore, a common sensor used for ball screw fault diagnosis is the accelerometer sensor (see Figure 5).

**Domain Gap**. In the fault diagnosis task of ball screws, the domain gap is mainly reflected in the differences in the data obtained by the sensors at different positions. Unlike rolling bearings, ball screw mechanisms have a more complicated motion trajectory in an operation cycle. Critical elements that need to be monitored, such as the ball screw nut assembly, are in motion when the ball screw is engaged. The ball screw nut also carries the work table on which workpieces are loaded, resulting in limited space available for sensor installation. One direction to solve this problem is to install the sensor on non-moving parts of the ball screw assembly, such as the front and end bearings, or in a location with proper space, such as the side of the table. However, due to the complex structure of the ball screw, the data collected by sensors placed in different locations vary greatly. Therefore, in the diagnosis of ball screw failure, the domain gap is mainly reflected in the difference in data collected by sensors in different positions.

### 3.2. Transportation

This section presents deep unsupervised domain adaptation of relevant components in transportation. This includes the capacity estimation of lithium-ion batteries, remaining useful lifetime estimation of turbofan engines and bearings, and fault diagnosis of the gearbox.

#### 3.2.1. Capacity Estimation of Lithium-Ion Batteries

**Background.** Due to their low self-discharge rate, low manufacturing cost, and high energy density [135], lithium-ion batteries have been widely deployed as energy storage devices in many fields, such as electronics and electric vehicles [136,137,138,139]. However, as the charge/discharge cycles increase, the performance of batteries will degrade due to the degradation of their electrochemical components. To ensure reliable operation and safety, it is important to estimate the capacity of individual cells online.

**Sensors.** The sensors commonly used in lithium-ion battery capacity estimation include current sensors, voltage sensors, and temperature sensors.

**Domain Gap.** In the actual operation of the battery system, different discharging and charging protocols will result in inconsistent capacity decay rates for different cells. This will result in significant differences in the data collected. In addition, due to individual differences in cell type or manufacture, batteries mostly show different degradation traces even under similar discharge/charge conditions.

**Datasets.** There are two commonly used public data sets for capacity estimation of lithium batteries, namely NASA Battery [64] and CALCE Battery [65].

(1) *NASA Battery Dataset* [64]. The NASA Battery Dataset is a dataset of lithium-ion batteries published by the NASA Ames Prognostics Center of Excellence. These batteries are commercially available 18650 lithium-ion batteries and operate through three different operating profiles (impedance, discharge, and charge) at different ambient temperatures. Repeated charge and discharge cycles result in accelerated aging of the batteries. Data collection was stopped when the batteries reached the end-of-life criteria of 30% fade in rated capacity.
(2) *CALCE Battery Dataset* [65]. The CALCE Battery dataset is a lithium-ion battery dataset presented by the Center for Advanced Life Cycle Engineering (CALCE) of the University of Maryland. These batteries undergo operations similar to NASA Battery, i.e., operation at different ambient temperatures through three different operating conditions (impedance, charging, and discharging). Each dataset contains five signals (i.e., discharge energy, internal resistance, voltage, current, and discharge time).

#### 3.2.2. Remaining Useful Lifetime Estimation

**Background.** Remaining Useful Lifetime (RUL) relates to the amount of time left before a piece of equipment is considered not to perform its intended function [77,140,141]. Accurate RUL prognostics enable assessing the health status of equipment and planning future maintenance actions.

**Sensors.** Common applications in RUL estimation include RUL estimation of turbofan engines and RUL estimation of bearings. Commonly used sensors for the former are temperature sensors, pressure sensors, flow sensors, etc. The latter commonly used sensors include acceleration sensors and temperature sensors.

**Domain Gap.** In the task of RUL estimation, what leads to the domain gap are the operating conditions and failure modes.

(1) *Different Failure Modes.* Taking RUL estimation of bearings as an example, the degradation of a bearing running to failure is usually associated with different failure modes, including inner failure, outer failure, etc., it is not a single failure behavior. Bearings in failure behavior under the action of the pressure and thermal strain usually lead to other failure modes. The degradation of bearings exhibits different behaviors due to different failure behaviors and, therefore, may result in differences in the distribution of features.
(2) *Different Operating Conditions.* In many practical applications, complete life degradation data are only available for bearings under certain operating conditions. However, the degradation process is often different when the operating conditions are different. Therefore, different operating conditions can also lead to a domain gap.

**Datasets.** Commonly used datasets in the estimation of RUL include the C-MAPPS dataset [68] and the IEEE PHM Challenge 2012 bearing dataset [69].

**Figure 6:**
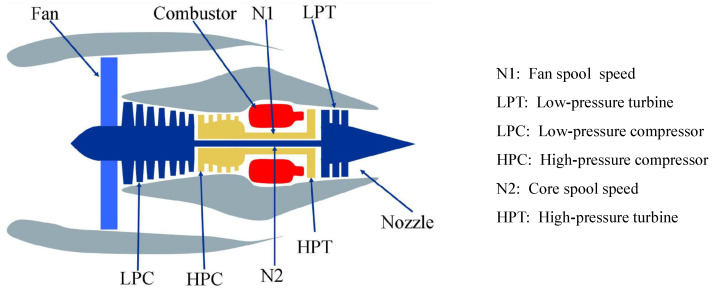
Diagram of the engines in C-MAPSS [68] dataset. Reprinted with permission from Ref. [68]. Copyright 2008 IEEE.

(1) *C-MAPPS* [68]. Commercial Modular Aero-Propulsion System Simulation (C-MAPPS) datasets contain degraded data for turbofan engines (see Figure 6) and consist of four different datasets, each containing information from 21 sensors and 3 operational settings. Each dataset has several degraded engines, divided into training and test instances. The engines in the datasets start with various degrees of initial wear but are considered healthy at the beginning of each record. As the number of cycles increases, the engines begin to deteriorate until they can no longer function. At this point, the engines are considered unhealthy. Training datasets collect run-to-failure information throughout their life cycle until failure. Unlike the training datasets, the test datasets contain temporal data that terminates some time before the system fails.
(2) *IEEE PHM Challenge 2012 bearing dataset* [69]. The IEEE PHM Challenge 2012 bearing dataset is collected from PRONOSTIA [69], which includes three key parts: the rotating part, load part, and data collection part. The platform provides the experimental run-to-failure data of rolling bearings through accelerated degradation experiments. Vibration and temperature signals are collected during all experiments. The frequency of vibration signal acquisition is 25.6 kHz. The frequency of temperature signal acquisition is 10 Hz. 600 samples are recorded each minute. The termination criterion of the experiment is set to a vibration amplitude of 20 g. The dataset is tested on 17 bearings under three different working conditions.

#### 3.2.3. Gearbox Fault Diagnosis

**Background**. In the mechanical transmission system, the gearbox is a frequently used transmission type that has been widely used in wind turbine [142,143], helicopter [144,145], automobile [146,147], et al. However, due to the harsh working conditions and complex meshing mode, gearboxes are easily subject to gear faults. The failure of the gearbox often leads to the failure of the whole mechanical system and brings great losses. Therefore, an effective and accurate fault diagnosis of the gearbox is of great importance in the field of transportation.

**Sensors.** In fault diagnosis of the gearbox, accelerometers are commonly used to obtain the vibration signal of the gearbox. Usually, one accelerometer is fixed in the *X*-axis direction and one in the *Y*-axis direction to obtain vibration information in different directions.

**Domain Gap.** The domain gap in gearbox fault diagnosis comes from different operating conditions, including speed and load. This frequent change in speed and load will greatly affect the domain distribution.

### 3.3. Biosignal

This section presents two research directions of UDA in biosignal, namely EEG-based brain-computer interface (BCI), human activity recognition (HAR), EMG-based muscle-computer interface (MCI), and gait analysis.

#### 3.3.1. EEG Based BCI

**Background.** Brain-computer interface is an important issue in biosignal and it enables users to communicate directly with computers using brain signals [97,98,148,149,150]. There are three general types of BCI, non-invasive BCI, invasive BCI, and partially invasive BCI. Electroencephalography (EEG) is an electrophysiological non-invasive technique for recording the electrical activity generated by the human brain, and it is increasingly being used in BCI tasks due to its safety, low cost and convenience.

**Sensors.** EEG signals usually are collected using a special device called an electroencephalogram. This device uses the principle of differential amplification or recording voltage differences between different points using a pair of special metal plate electrodes that compares one active exploring electrode site with another neighboring or distant reference electrode.

**Domain Gap.** The domain gap of EEG-based BCI is mainly reflected in different subjects and different sessions.

(1) *Different Subjects.* Brain signals show high variability among subjects due to inherent background neural activities, concentration levels, fatigue, etc. In this regard, deep learning-based BCI faces a great challenge in that classifiers trained on one subject cannot be directly used to decode brain signals from other subjects.
(2) *Different Sessions.* EEG signals are weak, susceptible to interference and noise contamination, nonstationary for the same subject, and varying across different sessions. Therefore, there are also differences in the distribution of signals collected at different sessions on the same subject.

**Datasets.** The common applications of EEG-based BCI are emotion recognition and motor imagery. The datasets related to emotion recognition include SEED [87] and DEAP [88]. The datasets related to motor imagery include BCI Competition IV-IIa [89] and BCI Competition IV-IIb [151].

**Figure 7:**
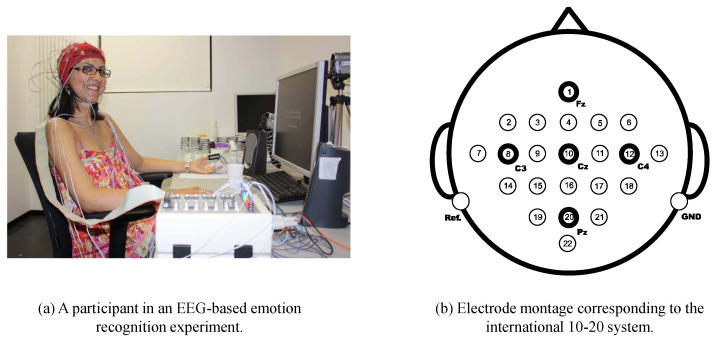
A participant in an EEG-based emotion recognition experiment [88] and electrode montage of BCI Competition IV-IIa [89]. Reprinted with permission from Ref. [88]. Copyright 2011 IEEE.

(1) *SEED* [87]. Shanghai JiaoTong University Emotion EEG Dataset (SEED) dataset consists of 15 participants, each of whom was required to watch 15 Chinese movie clips to induce three different kinds of emotions: negative, neutral, and positive. All of them are native Chinese with 7 males and 8 females. The movie clips are carefully selected as the stimuli for the experiment. All movies need to be comprehensible and neither too long nor too short to elicit sufficient emotional meaning. The EEG signals were recorded by an ESI NeuroScan System with a 62 electrode cap at a sampling rate of 1000 Hz.
(2) *DEAP* [88]. The Database for Emotion Analysis using Physiological Signals (DEAP) consisted of 32 subjects. Each subject was exposed to 40 1-min long music videos as emotional stimuli, while physiological signals were recorded. There were 40 EEG trials recorded for each subject, each corresponding to an emotion elicited by a music video. After watching each video, subjects were asked to assess the emotion they really felt in five ways: (1) familiarity (associated with the knowledge of the stimulus); (2) liking (associated with preference); (3) dominance (associated with control power); (4) arousal (associated with excitation level); and (5) valence (associated with pleasantness level). The scale ranges from 1 (weakest) to 9 (strongest). The EEG signals were recorded by Biosemi Active Two devices at a sampling rate of 512 Hz and downsampled to 128 Hz.
(3) *BCI Competition IV-IIa* [89]. The BCI Competition IV-IIa dataset (see Figure 7) consists of EEG signals of four different motor imagery tasks (left hand, right hand, foot, and tongue), which were acquired from nine subjects. According to the 10–20 system, all signals were recorded from 22 Ag/AgCl electrodes. The signals were sampled at 250 Hz and band-pass filtered between 0.5 Hz and 100 Hz.
(4) *BCI Competition IV-IIb* [151]. The BCI Competition IV-IIb dataset involves left-hand and right-hand motor imagery activity and contains three bipolar channel EEG signals from nine subjects. Each subject was sampled at a rate of 250 Hz and five time periods were collected.

#### 3.3.2. Human Activity Recognition

**Background.** Human activity recognition (HAR) is the recognition of user behavior from observed data collected by a set of sensors [152,153,154]. For example, inferring whether a user is standing or lying down based on data collected by accelerometers and gyroscopes embedded in a smartphone. HAR can be used for fall detection in the elderly, lifelogging systems for monitoring energy consumption, and digital assistants for weight lifting. As smart sensing technologies become more prevalent, more and more HAR systems are being deployed in our living environments. Therefore, accurate human activity recognition is of great importance to the biosignal field.

**Sensors.** In the task of human activity recognition, commonly used sensors are accelerometers and gyroscopes. There are also some recent studies [105,106] using wifi sensors for activity recognition.

**Domain Gap.** In the field of human activity recognition, domain gaps exist mainly in the differences between sensor signals acquired by different body parts of the same user and differences in sensor signals acquired by different users. Besides, there are also some differences in the signals acquired by different sensors, which can also lead to the domain shift of the model.

(1) *Different Body Parts*. Users often change the position of a sensor or wearable device based on their preferences and current activity. Despite doing the same activity, the sensor signals obtained from different body parts are very different. Therefore, transferring the activity model learned at one body location to another body location can reduce annotation costs and improve recognition accuracy and robustness to wearable device position changes.
(2) *Different Users*. Even when doing the same activity, there is some variation in the sensor signals collected by different users. A HAR system has a large number of users, and it is impractical or infeasible to collect enough ground truth from each user to build a model of the activity. Therefore, it is also very important to reduce the domain shift of the model between different users.
(3) *Different Sensors*. There are some differences in the data collected by different sensors (e.g., smartphones and smartwatches). Therefore, how to improve the performance of the model on newly deployed sensors without collecting any activity labels for new sensors is also a very important issue.
(4) *Different Environments*. When using WIFI sensors to identify human activities, different WIFI environments often result in biased data collection. For example, different indoor structures have different signal strengths and distributions of WIFI reflections [105]. Another example is that in in-vehicle human activity behavior recognition, different speeds of the car and interference from different wireless signals outside the car can lead to significant bias in the collected data [106].

**Datasets.** Common datasets in the field of human activity recognition include Opportunity [100], HHAR [101], PAMAP2 [102], and RealWorld [99].

**Figure 8:**
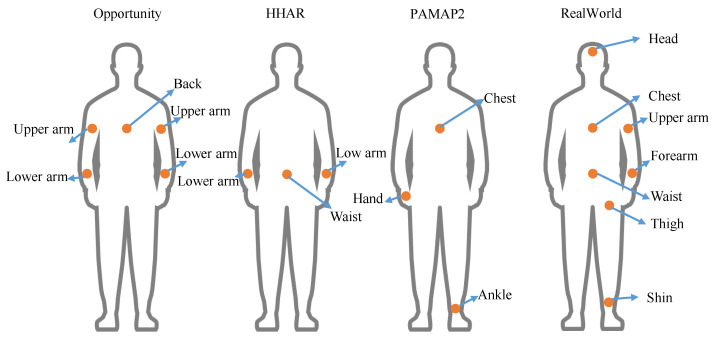
Placement of sensors in the Opportunity dataset [100], HHAR dataset [101], PAMAP2 dataset [102] and RealWorld dataset [99].

(1) *Opportunity* [100]. The Opportunity Activity Recognition dataset includes data recorded from 4 participants performing 5 activities: lying, sitting, walking, standing, and null. Users use custom-designed sports jackets and shoes to collect accelerometer signals from a total of 19 body positions. Accelerometer data were sampled simultaneously from the device at a sampling rate of 30 Hz. Users were asked to perform predefined daily home activities for 15–25 min. This is a challenging dataset since the sensors were placed on clothing rather than being tied to specific body locations.
(2) *HHAR* [101]. The Heterogeneity Human Activity Recognition (HHAR) dataset includes data recorded from 9 participants performing 6 activities: walk, stand, stairs up, stairs down, sit, and bike. Users were instrumented with 4 smartwatches and 8 smartphones. All 4 smartwatches were worn on each arm, and smartphones were placed around the waist. Accelerometer data were sampled simultaneously from the device at a sampling rate of 50 to 200 Hz. Each activity was performed for 5 min for each user. This dataset is noisy compared to the other datasets because the timestamps are not continuous and the sampling rate is not stable.
(3) *PAMAP2* [102]. The physical activity monitoring for aging people dataset 2 (PAMAP2) includes data recorded from 9 subjects performing 18 different activities, including ascending stairs, cycling, descending stairs, etc. Users used 3 wireless inertial measurement devices placed at 3 different body locations: head, chest, and ankle. Accelerometer and gyroscope data were sampled simultaneously from these devices at a sampling rate of 100 Hz. Each user performed up to 3 min of each activity.
(4) *RealWorld* [99]. The RealWorld HAR dataset includes data recorded from 15 participants performing 7 activities: walking, running/jogging, sitting, standing, climbing stairs down and up, lying, and jumping. The user places the smartphone and smartwatch in seven different body positions: head, chest, upper arm, waist, forearm, thigh, and calf. Accelerometer and gyroscope data are sampled simultaneously from the device at a sampling rate of 50 Hz. Figure 8 shows the location of the sensors in the different datasets.

#### 3.3.3. EMG Based MCI

**Background.** The muscle-computer interface (MCI) is an interaction method that translates myoelectrical signals directly from a mere reflection of muscle activity to an interactive command that conveys the intent of the user’s movement. It does not depend on actions performed by the user on a physical device, nor on externally visible or audible actions, thus enabling a readily available input mechanism in myoelectric control. As the technical core of non-invasive MCI, electromyography (EMG) technology is a technique that uses one or more electrodes to measure the electrical activity of muscles from the skin surface. Due to its safety and convenience, it is increasingly being used for MCI tasks.

**Sensors.** EMG signals are usually collected using a special device called an electromyogram. When muscles contract, they release a burst of electrical activity or electrical impulses that circulate through adjoining bones and tissues. The myoelectric potential is detected with surface and reference electrodes, and this potential is amplified using differential amplifiers to detect muscle or limb activation.

**Domain Gap.** The domain gap of EMG-based MCI is mainly reflected in different sessions and different subjects.

(1) *Different Sessions*. In practice, the electrode position may change when the EMG is taken off or put on, sweating, etc. This change is mainly caused by changes in electrode conductivity, electrophysiological changes, and electrode movement. This inherent non-stationarity leads to great variability in the signals collected by the EMG.
(2) *Different Subjects*. Since the EMG signal is a biological signal, the obtained signal varies considerably between subjects due to their physical condition. In this case, the variability of the data comes from the differences in the human body.

**Datasets.** Common datasets for EMG-based MCI include the CapgMyo dataset [111], the NinaPro dataset [112] and the CSL-HDEMG dataset [113].

**Figure 9:**
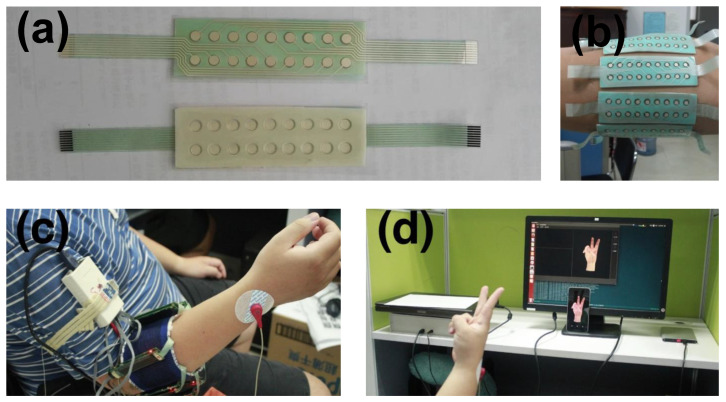
The acquisition setting-up for CapyMyo dataset [111]: (**a**) The EMG electrode array; (**b**) 8 EMG electrode arrays on the right forearm; (**c**) The EMG acquisition device ready for capture; (**d**) The software subsystem to present the guided hand gesture and record EMG data simultaneously.

(1) *CapgMyo* [111]. CapgMyo is an EMG-based gesture recognition dataset. It consists of 128 channels of HD EMG data acquired from 23 intact subjects with a sampling rate of 1 KHz. It consists of 3 sub-databases, DB-a, DB-b, and DB-c. DB-a contains 8 isometric, isotonic hand gestures obtained from 18 subjects. DB-b contains 8 isometric, isotonic hand gestures from 10 subjects in two recording sessions on different days. DB-c contains 12 basic movements of the fingers obtained from 10 subjects. Figure 9 illustrates the acquisition setup for the CapyMyo dataset.
(2) *NinaPro* [112]. The Ninapro (Non-Invasive Adaptive Prosthetics) dataset is a representative publicly available dataset in the field of surface myoelectricity research. Ninapro contains a total of 10 sub-datasets. Here, the DB-1 sub-dataset is introduced as an example. DB-1 sub-dataset is used to develop the hand prostheses and contains sparse multi-channel EMG recordings. It consisted of a total of 52 gestures performed by 27 intact subjects. Data are recorded at a sampling rate of 100 Hz, using 10 sparse electrodes placed on the subject’s upper forearm.
(3) *CSL-HDEMG* [113]. The CSL-HDEMG benchmark database is created specifically for EMG-based gesture recognition. EMG signals in CSL-HDEMG are recorded by using an electrode array with 192 electrodes covering the upper forearm muscles of five subjects performing a total of 27 gestures. The number of subjects in the CSL-HDEMG was relatively small compared to the CapgMyo dataset and the NinaPro dataset.

#### 3.3.4. Gait Analysis

**Background.** Walking and running are the most frequent movements in human life. Identifying gait phases is useful in a variety of applications. For example, physical therapists analyze gait phases to identify abnormal walking patterns. In sports science, gait phases are used to improve the skills and physical condition of athletes. Gait phases can also be used to synchronize the assistive forces of a wearable robot with the user’s movements.

**Sensors.** The sensors used for gait analysis include IMU sensors (including accelerometers and gyroscopes), infrared (IR) sensors, radar sensors, cameras, and EMG electrodes.

**Domain Gap.** For gait analysis, the domain gap mainly comes from the different positions of sensor placement, different subjects, and different moving states.

(1) *Different Positions of Sensors*. Such a problem is common in IMU sensor-based gait analysis. The position of the IMU may drift due to motion or personal preference, and it is difficult to precisely control the fixture of the IMU on users in real-world settings. Since IMU signals are location sensitive, the signals recorded at different locations can vary dramatically.
(2) *Different Subjects*. Human gait is highly individualized; each individual has his or her own unique gait pattern. Therefore, the sources of domain gaps in gait analysis also include different subjects.
(3) *Different Moving States*. In radar sensor-based gait analysis, differences in domains often stem from different states of motion. This is because when the test subject changes his or her motion state (different clothing, different motion speed, whether carrying an object, etc.), the radar echo intensity, Doppler bandwidth, or motion period will change. This also means that the distribution of gait data will also be shifted.

**Datasets.** Common gait analysis datasets include the Daphnet dataset [118], the OU-ISIR dataset [119], the CASIA-B dataset [120], and the CASIA-C dataset [121].

(1) *Daphnet* [118]. This dataset was designed to benchmark methods for automatically identifying gait freezes from wearable accelerometers placed on the legs and hips. The data was collected from 10 subjects with Parkinson’s disease using three wearable accelerometers. The three sensors were placed on the ankle (shank), thigh above the knee, and hip. Users performed three tasks: walking in a straight line, walking with multiple turns, and finally a more realistic activity of daily living task.
(2) *OU-ISIR* [119]. The OU-ISIR gait database was collected by the Institute of Scientific and Industrial Research (ISIR), Osaka University (OU). The dataset consists of people walking on the ground surrounded by 2 cameras at 30 fps and 640 × 480 pixels. These datasets are essentially distributed as silhouette sequences registered and size-normalized at 88 × 128 pixels.
(3) *CASIA-B* [120]. The CASIA-B dataset is published by the Institute of Automation, Chinese Academy of Sciences (CASIA). It contains 124 individuals captured from 11 viewpoints (0°, 18°, …, 180°). Each person walks six times in normal conditions, twice in their coats, and twice with bags to obtain a total of ten gait sequences. Overall, the CASIA-B dataset contains 13,640 sequences.
(4) *CASIA-C* [121]. The CASIA-C contains 1530 video clips, which are captured with a thermal infrared camera with a resolution of 320 × 240 pixels and a frame rate of 25 frames per second. Each video clip depicts a person walking in one of four different ways, including walking normally (normal), walking fast (fast), walking slowly (slow), and walking with a backpack (backpack).

## 4. Methods

In this section, unsupervised domain adaptation methods for time series sensor data are investigated. The most commonly used methods include adaptation in the input space, adaptation in the feature space, adaptation in the output space, and model-based adaptation (see Table 2).

In order to describe the different UDA methods more clearly, we have predefined some notations. Suppose the source domain $D_{S}$ is defined as follows:

(2) $$D_{s}={\left\{\left(x_{i}^{s},y_{i}^{s}\right)\right\}}_{i=1}^{n_{s}}\;x_{i}^{s}\in X_{s},\;y_{i}^{s}\in Y_{s},$$

where $x_{i}^{s}$ is the *i*-th sample as defined in Equation (1). $X_{s}$ is the union of all samples. $y_{i}^{s}$ is the *i*-th label of the *i*-th sample. $Y_{s}$ is the union of all different labels, and $n_{s}$ means the total number of source samples. The target domain is defined as:

(3) $$D_{t}={\left\{\left(x_{i}^{t}\right)\right\}}_{i=1}^{n_{t}}\;x_{i}^{t}\in X_{t},$$

where $x_{i}^{t}$ is the *i*-th sample. $X_{t}$ is the union of all samples. $n_{t}$ means the total number of target samples. The goal of unsupervised domain adaptation is to use the labeled source domain data $D_{s}$ and the unlabeled target domain data $D_{t}$ to improve the representation of the deep model in the target domain samples.

### 4.1. Adaptation in Input Space

Domain adaptation in the input space involves generating source domain samples that are very similar to the target domain, in the form of using generative adversarial networks or using prior knowledge. For example, in human behavior recognition, Sanabria et al. [104] use bi-directional generative adversarial networks [155] to generate source domain samples that are very similar to the target domain samples. Since the source domain samples are labeled, the performed transformed samples can be trained at scale using supervised learning, thus reducing the domain differences between the source and target domains. Another domain adaptation approach in the input space is to synthesize data artificially using a priori knowledge. For example, in fault diagnosis of bearings, Wang et al. [48] selects healthy real samples as the base signal for the synthesis process and uses expert knowledge to inject failure modes. Since the base signal encodes information about the operating and environmental conditions, the generated signal can be better adapted to the target domain, thus reducing the domain discrepancy.

### 4.2. Adaptation in Feature Space

**Figure 10:**
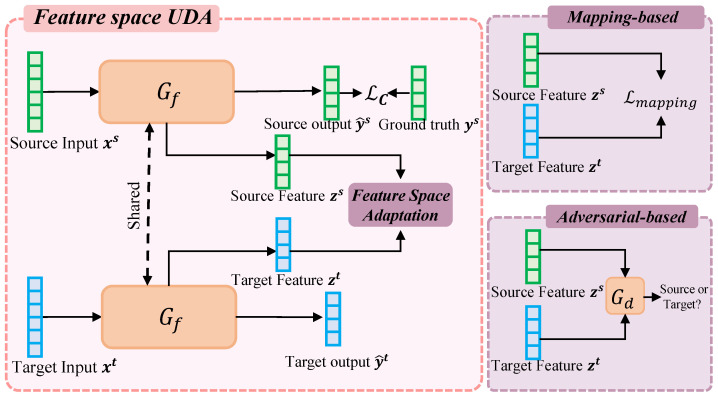
Diagram of feature space adaptation, including mapping-based (**top right**) and adversarial-based (**bottom right**) approaches.

Some domain adaptation methods unify the source and target domains by creating a domain-invariant feature representation, usually in the form of a feature extractor neural network. A feature representation is a domain invariant if the features follow the same distribution regardless of whether the input data is from the source or target domain. If a classifier can be trained to perform well on the source data using domain-invariant features, then the classifier generalizes well to the target domain because the features of the target data match the features for which the classifier is trained. Feature space adaptation methods typically include mapping-based methods and adversarial learning-based methods (see Figure 10). The next subsections will describe these two methods in detail.

#### 4.2.1. Mapping-Based Methods

The mapping-based approach includes mapping instances of the source and target domains to the feature space by a feature extractor and reducing the distance of the mapped features. Depending on the metrics, feature alignment methods include maximum mean discrepancy (MMD), multi-kernel MMD (MK-MMD), correlation alignment (CORAL), etc.

**MMD.** Maximum mean discrepancy is a two-sample statistical test of the hypothesis that two distributions are equally based on observed samples from the two distributions. The test is computed from the difference between the mean values of a smooth function on the samples from two domains. If the means are different, then the samples are most likely not from the same distribution. The smooth functions chosen for MMD are unit balls in feature reproducing kernel Hilbert spaces (RKHS) since it can be proven that the population MMD is zero if and only if the two distributions are equal. Assuming that the marginal distributions of the source and target domains are $P(X_{s})$ and $P(X_{t})$. The features extracted from the source domain sample $x^{s}$ and the target domain sample $x^{t}$ after passing through the network $G_{f}$ are $z^{s}$ and $z^{t}$, respectively. The naive MMD can be expressed as

(4) $$L_{\mathrm{mapping}}={∥E_{P}\left(\phi \left(z^{s}\right)\right)-E_{Q}\left(\phi \left(z^{t}\right)\right)∥}_{H_{k}}^{2},$$

where $H_{k}$ is RKHS using the kernel *k* (the Gaussian kernel is often used as the kernel) and ϕ(·) is the mapping to RKHS. Equation (4) makes the network extract similar features for the source and target domains, thus reducing the domain gap between them.

Suppose the predicted values of the source domain sample $x^{s}$ and the target domain sample $x^{t}$ after passing through the network $G_{f}$ are ${\hat{y}}_{i}^{s}$ and ${\hat{y}}_{i}^{t}$. The supervised learning loss of the source domain sample can be expressed:

(5) $$\begin{array}{c} L_{c}=-E_{\left(x_{i}^{s},y_{i}^{s}\right)\in D_{s}}\sum_{c=0}^{C-1}1_{\left[y_{i}^{s}=c\right]}\times \log{\hat{y}}_{i}^{s}, \end{array}$$

where *C* is the number of classes. Combining the Equation (4), the optimization objective of the network $G_{f}$ is

(6) $$\arg\min_{\theta_{f}}L_{c}+\lambda_{\mathrm{mapping}}L_{\mathrm{mapping}},$$

where $\theta_{f}$ is the parameter of the network $G_{f}$. $\lambda_{\mathrm{mapping}}$ is the balance parameter.

**MK-MMD.** The choice of parameters for each kernel is crucial to the final performance. Since different kernels have different performances, in practice, it is difficult to know which one to choose. To solve this problem, many scholars [57,70,78,81] have proposed to use MK-MMD, i.e., to build a total kernel with multiple kernels. It can maximize the testing power of two samples. For example, in the remaining useful lifetime estimation, Zhuang et al. [70] use MK-MMD to make the source domain features and the target domain features similar. In the fault diagnosis of bearings, Yang et al. [57] uses MK-MMD to reduce the difference between source and target domains among multiple features output from the network.

**CORAL.** The CORAL [60,84] is similar to MMD with a polynomial kernel and is computed from the distance between the second-order statistics (covariance) of the source and target features. For domain adaptation, the alignment component consists of computing the CORAL loss between the outputs of the two feature extractors. The CORAL loss function has applications in a wide range of industries. For example, Qin et al. [84] uses CORAL loss to improve the fault diagnostic capability of planetary gearboxes under different operating conditions. In fault diagnosis of power plant systems, Wang et al. [60] used CORAL loss to reduce the domain shift of the trained model.

#### 4.2.2. Adversarial-Based Methods

Adversarial-based methods refer to an adversarial approach that uses domain discriminators to reduce the difference in feature distribution between the source and target domains generated by the feature extractor. Like MMD and MK-MMD, adversarial-based methods are defined to solve the problem of unequal distributions. Unlike MMD and MK-MMD, adversarial-based methods do not use some metric to measure the distance between features directly, but introduce an additional discriminator $G_{d}$ (see Figure 10). The source and target domain features extracted by the feature extractor are input to the discriminator separately, and the parameters of the discriminator are optimized so that the discriminator can distinguish between them. The parameters of the feature extractor are optimized so that the discriminator is not able to distinguish between them. The formula is expressed as follows

(7) $$\begin{array}{c} L_{\mathrm{adv}}=E_{x_{i}^{s}\in D_{s}}\log\left[G_{d}\left(z_{i}^{s}\right)\right]+E_{x_{i}^{\prime }\in D_{t}}\log\left[1-G_{d}\left(z_{i}^{t}\right)\right]. \end{array}$$

Combining the supervised learning loss of the source domain samples in Equation (5), the optimization objective of the network $G_{f}$ can be expressed as

(8) $$\arg\min_{\theta_{f}}L_{c}+\lambda_{\mathrm{adv}}L_{\mathrm{adv}},$$

where $\lambda_{\mathrm{adv}}$ is the balance parameter. The objective optimization of discriminator $G_{d}$ can be expressed as

(9) $$\arg\max_{\theta_{d}}L_{\mathrm{adv}}.$$

By alternately optimizing Equations (8) and (9), the classification network $G_{f}$ is able to extract domain-invariant features, thus reducing the domain gap between the source and target domains.

Like mapping-based methods, there are a large number of methods in UDA that are based on adversarial learning. For example, Wang et al. [106] uses adversarial learning to improve the model’s ability to recognize human behavior. Zhang et al. [83] on the other hand, uses multilayer adversarial methods to improve the fault diagnosis of gearboxes. In the capacity estimation of lithium-ion batteries, Ye et al. [67] combines the strategies of MMD and adversarial learning to improve the performance of the model in the target domain.

### 4.3. Adaptation in Output Space

Pseudo-label-based domain adaptation methods [56,57,58] are the most common domain adaptation methods in the output space. Using classification as an example, the model’s confidence in different categories can be determined by looking at the softmax distribution in the last layer: uniformity indicates uncertainty, while a much higher probability for one category than the others indicates higher confidence. Applying this to domain adaptation, a diverse ensemble trained on the source data can be used to label the target data. These now-labeled target examples can then be used to train a classifier on the target data if the ensemble has a high level of confidence. This is thus a supervised process in the target domain, i.e., the network is trained using the assumed true labels (pseudo labels).

Pseudo-label-based domain adaptation methods also have some applications in solving the domain gap problem of time series sensor data. For example, Song et al. [56] uses a pseudo-label-based approach to do fault diagnosis of gearboxes. Yang et al. [57], on the other hand, combines MK-MMD and pseudo label to enhance the representation of the model under different operating conditions. Zhang et al. [58] proposes an iterative matching network enhanced with a selective sample reuse strategy. They use specially designed filters to select pseudo-label signals to increase the proportion of correctly labeled signals in the iterations and improve the performance of the model in the target domain.

### 4.4. Model-Based Adaptation Methods

The model-based domain adaptation approach is to reduce the domain shift of a model by imposing constraints on its parameters. For example, Jimenez et al. [91] use adaptive batch normalization [156] to align the domain distributions in terms of their first and second statistical moments. Khan et al. [110] proposes a transitional adaptation learning model specifically tuned to the properties of CNNs. They assume that the weight distribution in the convolutional layers remains essentially constant across contexts, and thus automatically adjusts the weights while minimizing the divergence of the weight distribution. Since the designed transfer learning method implicitly labels the activity of the source context, it does not require any explicitly labeled target training data to improve the performance of the model in the target domain.

## 5. Settings

**Figure 11:**
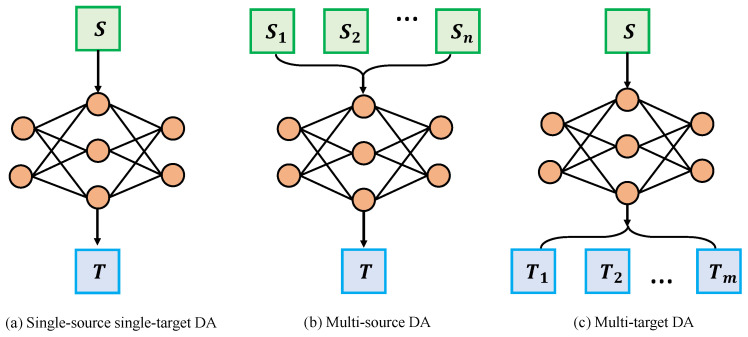
Different adaptation settings. *S* and *T* denote the source and target domains, respectively. $S_{1},S_{2},\cdots ,S_{n}$ and $T_{1},T_{2},\cdots ,T_{m}$ denote *n* different source domains and *m* different target domains, respectively.

This section describes the differences in adaptation settings caused by different numbers of domains in unsupervised domain adaptation, i.e., single-source single-target domain adaptation, multi-source single-target domain adaptation, and single-source multi-target domain adaptation. The differences between the different settings are shown in Figure 11 and Table 3. Each of the adaptation settings is described in detail next.

### 5.1. Single-Source Single-Target Domain Adaptation

The vast majority of current domain adaptation algorithms focus on a single-source single-target setup. This setup is relatively simple, assuming only one source and one target domain. The single domain gap motivates a large number of approaches, including domain adaptation in the input space, domain adaptation in the feature space, etc. However, this single-source single-target setup can have limitations. In reality, the sensor deployment environment is always changing, and models adapted in a single target domain are difficult to transfer to other target domains (data collected under changing sensor deployment conditions). Moreover, during domain adaptation, the available supervised samples may come from multiple source domains with different distributions. Therefore, it is also important to utilize multiple source domains with labels to improve the performance of the target domain.

### 5.2. Multi-Source Domain Adaptation

Unlike single-source domain adaptation, multi-source domain adaptation can obtain more comprehensive knowledge by fusing supervised samples from multiple source domains and mitigate the risk of overfitting problems by having more training samples, which is beneficial to improving the performance of the target domain.

In order to effectively fuse different source domains, Zhu et al. [45] proposes a multiple adversarial learning strategy with multiple domain discriminators to learn domain invariant and discriminative features of the task. Through the multi-adversarial learning strategy, the samples from different rolling bearings are projected into a shared feature subspace so that domain invariant features are obtained. Meanwhile, the extracted features are also task-discriminative since sufficient labeled samples in the source domains are available. Xia et al. [41], on the other hand, proposes a novel multi-source domain adaptation model, which uses a feature learner to generate features for each source and target domain data to enable a joint weight classifier to predict the target labels. They also introduce an MMD-based distance metric to reduce the distance between all source and target domains. In the training process of the model, an intra-class matching training strategy is used to match the distribution of each domain to improve the recognition accuracy of the target domain. In the field of human activity recognition, Chakma et al. [108] proposes a deep multi-source adversarial domain adaptation framework that opportunistically helps select the most relevant feature representations from multiple source domains and establish such mappings to the target domain by learning the perplexity scores.

Although multi-source domain adaptation achieves better results compared to single source domain adaptation, this setting may lead to negative adaptation, i.e., some source domains may not have a positive effect on improving the model’s performance in the target domain. To address this issue, Wei et al. [55] proposes a multi-source domain adaptation model for weighting different source domains. In particular, they insert a weighting module in the output space to avoid negative adaptation of source domains by adaptively controlling the weights of different source domains.

### 5.3. Multi-Target Domain Adaptation

In practical application scenarios, different working conditions and different environments result in different domain gaps. Therefore, it is important to train a model to have a good performance on multiple target domains at the same time. There are two naive ways of directly extending domain-specialized UDA to work on multiple target domains, that are (1) training a single model on combined data from multiple target domains, and (2) training multiple models individually for each target domain. Unfortunately, these methods are not appropriate to handle multi-target domain adaptation problems because they would suffer from performance degradation due to the mismatching of multi-target domains [157].

How to explore the potential connection between multiple target domains is an important research topic. There is very limited research on multi-target-based domain adaptation in time series sensor data. Ref. [50] proposes a framework for single-source to multi-target domains for bearing fault diagnosis. They first train the source feature extractor to obtain class discriminative features using the labeled source domain. Then, the target feature extractors are initialized by the weights of the source feature extractor and, thus, inherit the class-discriminative property. On the other hand, a discriminator network is trained to distinguish between the source and multi-target features. To obtain domain-invariant features among different targets, They adversarially update multi-target feature extractors to generate features that can be indistinguishable for the discriminator. During testing, the scalable model can take any of the target domains and generate source-like features, where the trained source classifier is able to generalize well to any of the targets.

## 6. Discussion

As shown in Section 3, UDA technology is heavily used in a variety of industries due to the complex environment in the real world. Nevertheless, there are still some potential research directions worth exploring. For example, with the popularity of smart wearable devices, more and more manufacturers are using smart devices to obtain the user’s heart rate and electrocardiogram to measure the user’s physical condition. However, models trained on some people do not generalize well to others due to the differences in fitness of different people. Therefore, how to reduce the domain shift of heart rate recognition models or electrocardiogram recognition models is a problem worth investigating.

In addition to the applications of UDA, there will be some room for exploration in the UDA approach. Most of the deep models used by existing methods are CNN and RNN, and if the backbone network is replaced with Transformer [30] that deals with time series sensor data, it may also improve the adaptation performance. Another way is to use knowledge distillation [158] to train the teacher in the source domain, and combine it with pseudo label generation to teach the student in the target domain, and improve the performance through continuous iteration. Similarly, for the multi-source domain adaptation approach, multiple teachers can be used, each with a different direction of focus. In this way, it may be possible to better transfer models trained with data from different source domains to the target domain, thus improving the performance in the target domain. Another strategy that can be explored for unsupervised domain adaptation is to use contrast learning [159]. That is, a large amount of unlabeled data is used to enhance the ability of the model to acquire features through contrast learning, and then traditional unsupervised domain adaptation methods are used to make the features in the source and target domains close in distribution, and thus reduce the domain differences between the source and target domains.

In terms of UDA settings, existing methods rarely explore multi-source to multi-target domain adaptation. This adaptation setting would include the advantages of both multi-source adaptation and multi-target adaptation, i.e., the source domain contains data collected under multiple conditions and can simultaneously contribute to improving the model performance in the target domain. Also, since multiple target domains are included, the adapted model can be generalized to multiple operating conditions.

## 7. Conclusions

Complex environments and variable working conditions result in deep learning models trained with time series sensor data collected in one condition performing poorly when tested in another. In recent years, more and more scholars have adopted the unsupervised domain adaptation approach to solve the domain shift problem of deep models. This survey systematically studied, analyzed and summarized the unsupervised domain adaptation technique based on time series sensor data from three aspects: application, method, and setup. In particular, this survey analyzed and summarized which sensors are used in different application directions, where the domain gaps are, and what data are publicly available. Moreover, this survey classified and compared different unsupervised domain adaptation methods and discussed and summarized the advantages of various settings. This survey also provided an outlook on potential application areas, explored feasible research directions, and discussed additional settings for unsupervised domain adaptation. Through this survey, we hope to provide a comprehensive and systematic understanding to readers who are investigating the direction of unsupervised domain adaptation with time series sensor data.

## Figures and Tables

**Table 1:** Classification results based on different applications.

	Applications	Sensors	Domain Gap	Datasets	References
Industry	fault diagnosis of rolling bearings	accelerometer, microphone	different working conditions, different positions of the sensors, different machines	CWRU [38], PU [39], IMS [40]	[41,42,43,44,45,46,47,48,49,50,51,52,53,54,55,56,57,58,59]
	fault diagnosis of power plant thermal system	temperature sensors, pressure sensors, flow rate sensors, etc.	different fault severity, different load conditions	-	[60,61]
	diagnosis of ball screw failure	accelerometer	different positions of the sensors	-	[62,63]
Transportation	capacity estimation of lithium-ion batteries	current sensor, voltage sensor, temperature sensor	different charging/discharging protocols, different in cell type and manufacture	NASA Battery [64], CALCE Battery [65]	[66,67]
	remaining useful life estimation	accelerometer, temperature sensor, pressure sensor, flow rate sensor, etc.	different operating conditions, different failure modes	C-MAPPS [68], IEEE PHM Challenge 2012 bearing dataset [69]	[70,71,72,73,74,75,76,77,78,79,80,81]
	gearbox fault diagnosis	accelerometer	different operating conditions	-	[82,83,84,85,86]
Biosignal	EEG based brain-computer interface	EEG electrodes	different subjects, different sessions	SEED [87], DEAP [88], BCI Competition IV-IIa [89], BCI Competition IV-IIb [90]	[91,92,93,94,95,96,97,98]
	human activity recognition	accelerometers, gyroscopes, wifi sensor	different body parts, different users, different sensors	RealWorld [99], Opportunity [100], HHAR [101], PAMAP2 [102]	[103,104,105,106,107,108,109,110]
	EMG based muscle-computer interface	EMG electrodes	different sessions, different subjects	CapgMyo [111], NinaPro [112], CSL-HDEMG [113]	[111,114,115,116,117]
	gait analysis	accelerometers and gyroscopes, IR sensors, radar, camera, EMG electrodes	different positions of sensors, different subjects, different moving states	Daphnet [118], OU-ISIR [119], CASIA-B [120], CASIA-C [121]	[122,123,124,125,126,127]

**Table 2:** The classification results based on different domain adaptation methods.

Different Adaptation Methods	Description	References
Input space	By generating source domain samples that are very similar to the target domain, the gap in the source domain is reduced by supervised training.	[48,104,109]
Feature Space (mapping-based)	The features are mapped to some space and then a metric is used to reduce the distance between the source and target domains.	[41,44,46,47,51,52,53,54,59,60,61,62,63,66,67,70,71,73,76,78,79,81,82,83,84,86,91,103,104,107,122,123,127]
Feature Space (adversarial-based)	The discriminator is used to identify whether the generated features are from the source or target domain. The feature extraction network tries to fool the discriminator. As a result, the network is able to generate similar features for both source and target domain samples.	[42,43,45,49,50,55,67,71,72,74,75,76,77,80,81,84,85,92,93,94,95,96,105,106,108,124,126,96]
Output space	The labels with high confidence are selected for the target domain, and these pseudo-labels are used to do supervised training on the target domain samples.	[56,57,58,114]
Model-based	By constraining the parameters of the model, the model can be adapted to the sample of the target domain.	[91,110,111,115,116,117,125]

**Table 3:** Classification results based on the number of source and target domains.

Different Settings of Domain Adaptation	Advantages	References
Single-source single-target domain adaptation	This setup is simple and more focused on the target area.	[41,42,46,47,48,49,51,53,54,55,56,57,58,59,60,61,62,63,66,67,70,71,72,73,74,75,76,77,78,79,80,81,82,83,84,85,86,91,92,93,95,96,103,104,106,107,109,110,114,115,116,123,125,126]
Multi-source domain adaptation	Each source domain has its own focus and can integrate different aspects of information.	[43,44,45,52,94,105,108,111,117,124,127]
Multi-target domain adaptation	The trained model can be adapted to multiple working conditions simultaneously.	[50,122]

## Data Availability

No new data were created or analyzed in this study. Data sharing is not applicable to this article.

## Abbreviations

The following abbreviations are used in this manuscript:

CNN	Convolutional Neural Network
RNN	Recurrent Neural Network
LSTM	Long Short-Term Memory
GRU	Gated Recurrent Unit
UDA	Unsupervised Domain Adaptation
CWRU	Case Western Reserve University
PU	Paderborn University
CALCE	Center for Advanced Life Cycle Engineerin
RUL	Remaining Useful Lifetime
C-MAPPS	Commercial Modular Aero-Propulsion System Simulation
EEG	Electroencephalogram
BCI	Brain–Computer Interface
HAR	Human Activity Recognition
SEED	Shanghai JiaoTong University Emotion EEG Dataset
DEAP	Database for Emotion Analysis using Physiological Signals
HHAR	Heterogeneity Human Activity Recognition
RKHS	Reproducing Kernel Hilbert Spaces
MMD	Maximum Mean Discrepancy
MK-MMD	Multi-kernel Maximum Mean Discrepancy
CORAL	Correlation Alignment
PAMAP	Physical Activity Monitoring for Aging People
CASIA	Institute of Automation, Chinese Academy of Science
OU-ISIR	Institute of Scientific and Industrial Research, Osaka University
MCI	Muscle-Computer Interface
EMG	Electromyograph
IMS	Intelligent Maintenance System
